# Molecular Analyses Define Vα7.2-Jα33+ MAIT Cell Depletion in HIV Infection: A Case–Control Study

**DOI:** 10.1097/MD.0000000000001134

**Published:** 2015-07-24

**Authors:** James E. Ussher, Prabhjeet Phalora, Cormac Cosgrove, Rachel F. Hannaway, Andri Rauch, Huldrych F. Günthard, Philip Goulder, Rodney E. Phillips, Christian B. Willberg, Paul Klenerman

**Affiliations:** From the Peter Medawar Building for Pathogen Research (JEU, PP, CC, PG, REP, CBW, PK), University of Oxford, Oxford, UK; Department of Microbiology and Immunology (JEU, RFH), University of Otago, Dunedin, New Zealand; Ragon Institute of MGH, MIT and Harvard (CC), Cambridge, Massachusetts, USA; Division of Infectious Diseases (AR), University Hospital Berne and University of Berne, Berne; Division of Infectious Diseases and Hospital Epidemiology and Institute of Medical Virology (HFG), University Hospital Zurich, University of Zurich, Zurich, Switzerland.

## Abstract

Mucosal-associated invariant T (MAIT) cells are an abundant antibacterial innate-like lymphocyte population. There are conflicting reports as to their fate in HIV infection. The objective of this study was to determine whether MAIT cells are truly depleted in HIV infection.

In this case-control study of HIV-positive patients and healthy controls, quantitative real-time polymerase chain reaction was used to assess the abundance of messenger RNA (mRNA) and genomic DNA (gDNA) encoding the canonical MAIT cell T cell receptor (Vα7.2-Jα33). Comparison was made with flow cytometry.

Significant depletion of both Vα7.2-Jα33 mRNA and gDNA was seen in HIV infection. Depletion of Vα7.2+CD161++ T cells was confirmed by flow cytometry. In HIV infection, the abundance of Vα7.2-Jα33 mRNA correlated most strongly with the frequency of Vα7.2+CD161++ cells. No increase was observed in the frequency of Vα7.2+CD161− cells among CD3+CD4− lymphocytes.

MAIT cells are depleted from blood in HIV infection as confirmed by independent assays. Significant accumulation of a CD161− MAIT cell population is unlikely. Molecular approaches represent a suitable alternative to flow cytometry-based assays for tracking of MAIT cells in HIV and other settings.

## Abstract 2

Supplemental Digital Content is available in the text

## INTRODUCTION

Mucosal-associated invariant T (MAIT) cells are innate-like T cells that comprise ∼5% of the T-cell population in adult blood and are further enriched in mucosal and liver tissues.^1–3^ MAIT cells are restricted by the nonpolymorphic, highly evolutionarily conserved major histocompatibility complex, class Ib-related protein (MR1).^2^ MR1 has recently been shown to present a metabolite derived from the riboflavin synthetic pathway.^4,5^ Consistent with this, MAIT cells are activated by riboflavin-producing bacteria, including *Mycobacterium tuberculosis*, *Escherichia coli*, and *Salmonella* species.^6,7^ MAIT cells have also been shown to protect against bacterial infection in vivo, including against dissemination of bacillus Calmette–Guerin (BCG) in a mouse model.^8^

MAIT cells have a semiinvariant T-cell receptor (TCR), Vα7.2-Jα33, and utilize a limited range of Vβ chains.^9^ Additional minor MR1-restricted MAIT cell populations with Vα7.2-Jα20 and Vα7.2-Jα12 have recently been described through use of an MR1 tetramer.^10^ MAIT cells are defined by high levels of expression of the C-type lectin, CD161.^3^ More than 90% of MAIT cells are CD8α+, whereas ∼7% are CD4/CD8 double negative and <1% are CD4+.^11^ MAIT cells also express high levels of the interleukin (IL)-18 receptor and are able to make interferon-γ in response to IL-12 and IL-18 in the absence of TCR stimulation.^12^

Recently, several reports have identified a significant impact of HIV infection on MAIT cells.^13–18^ Although all studies show a loss of CD161++ Vα7.2+ MAIT cells, it has been suggested that MAIT cells downregulate CD161 after activation and that in HIV infection the frequency of MAIT cells (including both CD161++Vα7.2+ and CD161−Vα7.2+ MAIT cells) is unchanged.^14^ Importantly, the Vα7.2 antibody (clone 3C10) used in all these studies^13–18^ is not specific for the canonical MAIT cell TCR, but in combination with CD161 expression accurately identifies MAIT cells.^3^ Therefore, we sought to determine whether MAIT cells were preserved or depleted in HIV through quantitative real-time polymerase chain reaction (PCR) for the canonical MAIT cell TCR, Vα7.2-Jα33.

## METHODS

### Blood Samples

Leukocyte cones from unidentified healthy controls (HCs) (n = 38) were obtained from NHS Blood and Transplant. Blood was obtained from HIV-infected patients (HIV+) in the Thames Valley (n = 26)^19^ and Swiss HIV cohorts (n = 20) or HCs (n = 12). Peripheral blood mononuclear cells (PBMCs) were purified on Lymphoprep gradients,^13^ and cryopreserved prior to analysis. Clinical details of patients in the Thames Valley cohort are shown in Table 1. Clinical details of the patients in the Swiss HIV cohort have previously been reported,^13^ and are re-presented here in Table 1.

**TABLE 1:**
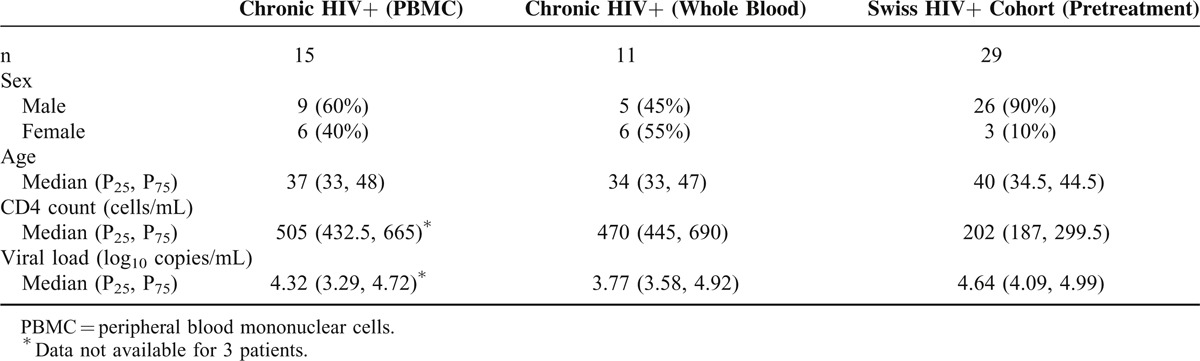
Clinical Characteristics of HIV+ Cohorts

Written, informed consent was given by participants. The collection of samples was approved by the relevant ethics committees (the Central University Research Ethics Committee at the University of Oxford and the relevant ethics committees at all the participating institutions in Switzerland).

### Quantitative Real-Time Reverse Transcription-PCR

RNA was extracted from PBMCs (13 HCs, 15 HIV+) using the MiniRNA extraction kit (Qiagen, Hilden, Germany) or, in sorting experiments, the NucleoSpin RNA isolation kit (Machery Nagel, Bethlehem, PA, USA), as per the manufacturer's instructions with on-column DNase digestion. Reverse transcription was performed with Superscript III using a mix of anchored oligo(dT)20 primers (0.625 μg) and random primers (2.25 μg) (all Life Technologies, Carlsbad, CA, USA) as per the manufacturer's instructions. Samples were then incubated with 2U of RNAseH (Life Technologies, Carlsbad, CA, USA) for 20 minutes at 37^o^C before use in a real-time PCR. In sorting experiments, high-capacity RNA-to-cDNA kit (Life Technologies, Carlsbad, CA, USA) was used as per the manufacturer's instructions.

### Genomic DNA Extraction

Genomic DNA (gDNA) was extracted from whole blood (12 HCs, 11 HIV+) or PBMCs (10 HCs) as previously described.^20^ The same volume of eluted DNA extracted from the same volume of blood was used for quantitative real-time PCR. In sorting experiments, the PureLink Genomic DNA Mini Kit (Life Technologies, Carlsbad, CA, USA) was used.

### Quantitative Real-Time PCR

Quantitative PCR was performed on complementary DNA or gDNA. Vα7.2-Jα33 and Cα were amplified in singleplex reactions using previously described primers (Life Technologies, Carlsbad, CA, USA) and Taqman probes (Fluorophore: 6FAM, Quencher: BQX [Sigma-Aldrich, St Louis, MO, USA]).^9^ For sorting experiments, the β2-microglobulin (B2M) forward primer (GTGCTGTCTCCATGTTTGATG), reverse primer (TCTGCTCCCCACCTCTAAG), and probe (6-FAM/AGGTTGCTC/ZEN/CACAGGTAGCTCTAGG/Iowa Black FQ) (all Integrated DNA Technologies, Coralville, IA, USA) were used.

Briefly, primers were added at a final concentration of 200 nM, Taqman probes at 250 nM, and FastStart Universal Probe Mastermix (Roche, Mannheim, Germany) or, in sorting experiments, KAPA PROBE FAST qPCR Master Mix (2X) Universal Kit (Kapa Biosystems, Wilmington, MA, USA) was used. PCR was performed on a LC480 (Roche, Mannheim, Germany) or, in sorting experiments, an ABI 7500 Fast (Applied Biosystems, Foster City, CA, USA), using the following cycling conditions: 95^o^C for 5 minutes, then 40 cycles of 94^o^C for 10 seconds, 58^o^C for 30 seconds, and 72^o^C for 10 seconds, then 40°C for 10 seconds. C_P_/C_T_ was automatically determined by the instrument's software. The expression of Vα7.2-Jα33 relative to Cα (or B2M) was determined by the comparative C_T_ method (2^ΔΔCT^). The efficiencies of the various PCR reactions are shown in the Supplementary Figure 1, http://links.lww.com/MD/A337.

### Flow Cytometry

The following antibodies were used: Vα7.2-PE, CD3-PECy7 (BioLegend, San Diego, CA, USA), CD4-VioGreen, CD161-APC (Miltenyi Biotec, Bergisch Gladbach, Germany), CD4-APCeFluor780, CD8-eFluor450 (eBiosciences, San Diego, CA, USA), CD3-Pacific Orange (Life Technologies, Carlsbad, CA, USA), CD8-PECy7 (BD Biosciences, San Jose, CA, USA). All samples were stained with Live/Dead Fixable Near IR dye (Life Technologies, Carlsbad, CA, USA). Samples were stained as previously described.^13^ Flow cytometry was performed on a MACSQuant (Miltenyi Biotec, Bergisch Gladbach, Germany), an LSRII (BD Biosciences, San Jose, CA, USA), or for sorting experiments, on a FACSAria (BD Biosciences, San Jose, CA, USA). Samples were gated on lymphocytes/alive/CD3+ or lymphoctyes/alive/CD3+/CD4− (Supplementary Figure 2, http://links.lww.com/MD/A337). Analysis was performed in FlowJo 9.6 (Treestar, Inc., San Carlos, CA, USA).

### Statistical Analysis

No formal power calculation was performed. Data were analyzed in Prism 6 (GraphPad Software, San Diego, CA, USA). Medians and all data points are shown. Comparisons between groups were made by *t* tests with log_10_-normalized data. For comparison of C_T_ values, nonnormalized data was used. For sorting experiments, a paired *t* test on log_10_ normalized data was performed. Spearman correlations were calculated separately for HIV+ and HC cohorts. Significance was defined as 2-sided *P* ≤ 0.05.

## RESULTS

**FIGURE 1:**
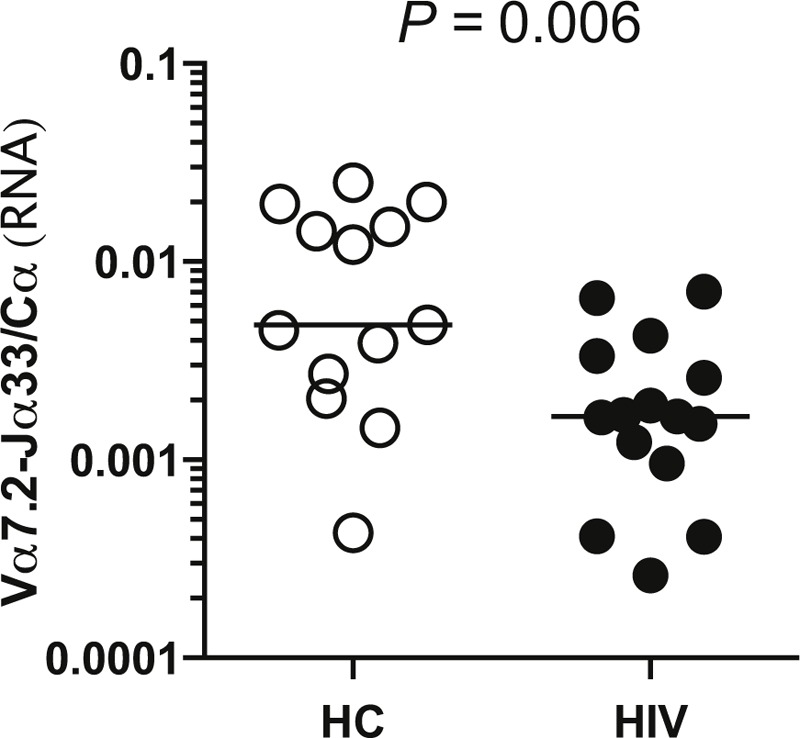
Depletion of Vα7.2-Jα33 messenger RNA from the blood in HIV infection. The frequency of MAIT cells in blood from HCs (open circles) or HIV-infected patients (filled circles) was determined by quantitative real-time reverse transcription PCR. HC = healthy control, MAIT = mucosal-associated invariant T, PCR = polymerase chain reaction.

The Vα7.2 antibody not only recognizes MAIT cells, which express the canonical Vα7.2-Jα33 TCR, but also other non-MAIT cell TCRs. Therefore, we sought to determine the number of MAIT cells in blood from HIV-infected and HIV-uninfected donors by quantitative real-time reverse transcription (RT)-PCR. The expression of Vα7.2-Jα33 relative to the Cα chain, which is expressed in all αβ T cells, was determined. Vα7.2-Jα33 messenger RNA (mRNA) was significantly less abundant in the blood of patients with HIV than uninfected controls (median relative expression of Vα7.2-Jα33:Cα 0.00165 vs 0.00478, *P* = 0.006) (Figure 1).

**FIGURE 2:**
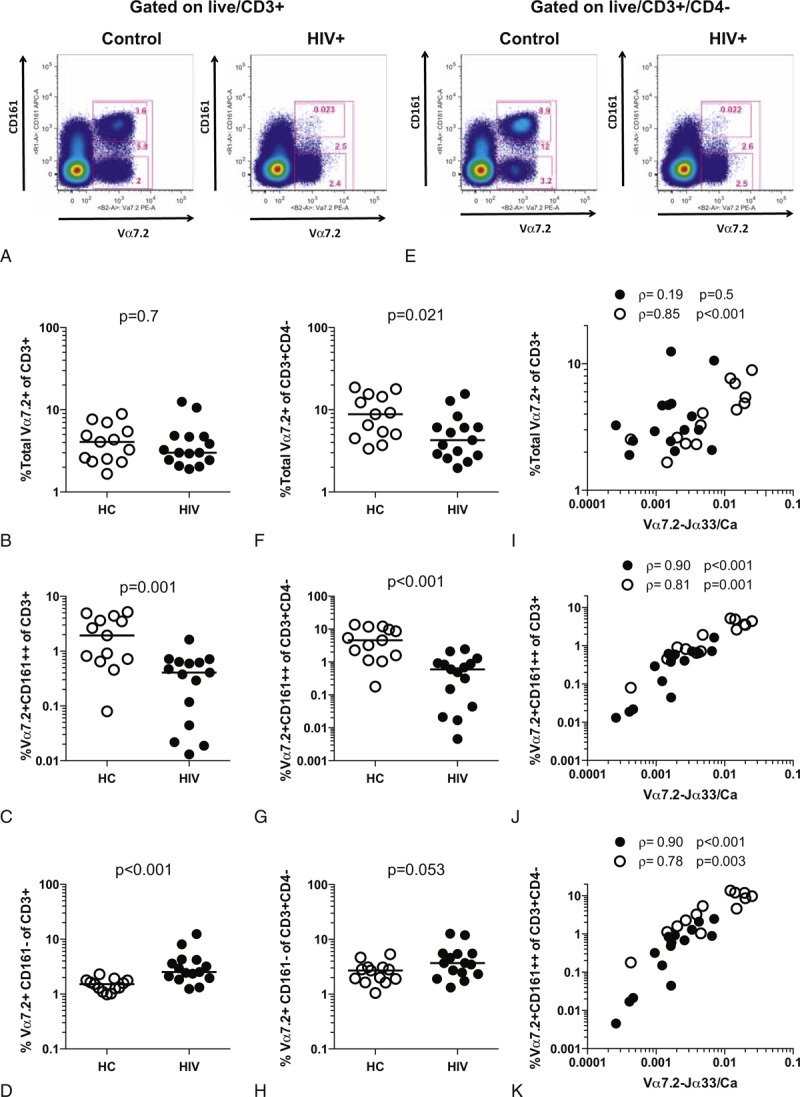
MAIT cells are depleted in the blood of HIV-infected patients. The frequency of MAIT cells in blood from HCs (open circles) or HIV-infected patients (filled circles) was determined by flow cytometry. (A, E) Representative plots and (B, F) cumulative data of the relative frequencies of the Vα7.2+, (C, G) Vα7.2+CD161++, and (D, H) Vα7.2+CD161− populations among (A–D) CD3+ lymphocytes and (E–H) CD3+CD4− lymphocytes in HC and HIV-infected patients is shown. Correlation of the frequency of (I) Vα7.2+CD3+ cells, (J) Vα7.2+CD161++CD3+ cells, or (K) Vα7.2+CD161++CD3+CD4− cells, as assessed by flow cytometry, with the abundance of MAIT cells, as assessed by quantitative real-time RT-PCR. Spearman correlations were calculated separately for HIV-infected patients and for HCs. The same samples were analyzed in Figures 1 and 2. HC = healthy control, MAIT = mucosal-associated invariant T, RT-PCR = reverse transcription polymerase chain reaction.

To confirm MAIT cell depletion at the cellular level, the same PBMCs were analyzed by flow cytometry. Frequencies were determined as a proportion of CD3+ lymphocytes (Figure 2A). No difference in frequency of Vα7.2+ T cells was seen (Figure 2B). Vα7.2+CD161++ cells were depleted (0.41% vs 1.93%, *P* = 0.001) (Figure 2C) whereas Vα7.2+CD161− cells were increased (2.52% vs 1.52%, *P* < 0.001) in HIV-infected patients compared with HCs (Figure 2D).

As depletion of CD4+ T cells in HIV distorts relative frequencies of other populations, and <1% of MAIT cells are CD4+,^11^ frequencies were also determined as a proportion of CD3+CD4− lymphocytes (Figure 2E). Vα7.2+ cells were depleted in HIV-infected patients compared with HCs as a proportion of CD3+CD4− lymphocytes (4.27% vs 8.82%, *P* = 0.021) (Figure 2F). In HIV-infected patients versus HC, Vα7.2+CD161++ cells were significantly depleted (0.60% vs 4.59%, *P* < 0.001) (Figure 2G), but no significant increase in Vα7.2+CD161− cells was seen (3.69% vs 2.7%, *P* = 0.053) (Figure 2H).

Next we determined which flow cytometric measure best correlated with the frequency of MAIT cells, as determined by quantitative RT-PCR. When CD4+ cells were included, there was no significant correlation between Vα7.2-Jα33 mRNA levels and frequency of Vα7.2+ T cells (ρ = 0.19, *P* = 0.49) in HIV-infected patients (Figure 2I). In contrast, Vα7.2-Jα33 mRNA levels in HCs were significantly correlated with frequency of Vα7.2+ T cells (ρ = 0.85, *P* < 0.001) (Figure 2I). The frequency of Vα7.2+CD161++CD3+ cells significantly correlated with Vα7.2-Jα33 mRNA levels in HIV-infected patients (ρ = 0.90, *P* < 0.001) and HCs (ρ = 0.81, *P* = 0.001) (Figure 2J).

When CD4+ cells were excluded, the frequency of Vα7.2+ cells among CD3+CD4- lymphocytes correlated significantly with Vα7.2-Jα33 mRNA levels in HC (ρ = 0.78, p = 0.003) but not HIV-infected patients (ρ = 0.38, p = 0.17) (data not shown). The frequency of Vα7.2+CD161++ cells amongst CD3+CD4- lymphocytes correlated significantly with Vα7.2-Jα33 mRNA levels in both groups (HIV+: ρ = 0.90, p < 0.001; HC: ρ = 0.78, p = 0.003) (Figure 2K). Therefore, in HIV infection frequency of Vα7.2+CD161++ cells most accurately reflects abundance of MAIT cells.

**FIGURE 3:**
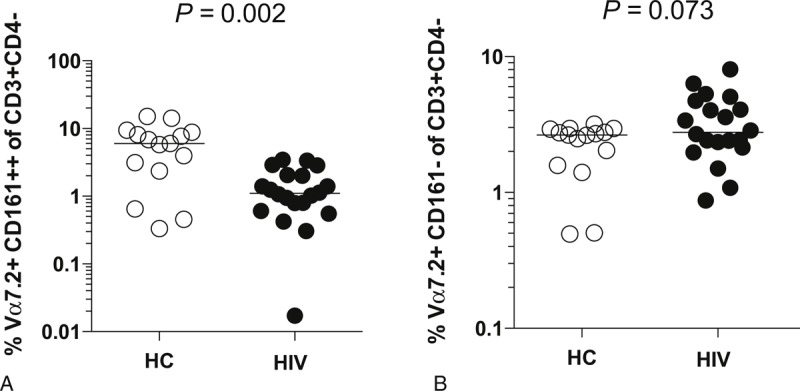
MAIT cells are depleted in the blood of HIV-infected patients: Swiss HIV Cohort. The frequency of MAIT cells in blood from HCs (open circles) or HIV-infected patients (filled circles) was determined by flow cytometry. Cumulative data of the relative frequencies of the (A) Vα7.2+CD161++ and (B) Vα7.2+CD161− populations among CD3+CD4− lymphocytes in HCs and HIV-infected patients is shown. HC = healthy control, MAIT = mucosal-associated invariant T.

To confirm that MAIT cells are depleted in HIV infection and that Vα7.2+CD161− cells are not significantly enriched, we reanalyzed our previously published data from the Swiss HIV Cohort Study excluding CD4+ T cells.^13^ Comparison of the pretreatment timepoint with HCs demonstrated a significant depletion of Vα7.2+CD161++ cells as a proportion of CD3+CD4− lymphocytes (1.10% vs 4.59% in HIV+ vs HC, *P* = 0.002) (Figure 3A). No significant difference was seen in the proportion of Vα7.2+CD161− cells (median 2.77% vs 2.65% in HIV+ vs HC, *P* = 0.073) (Figure 3B).

**FIGURE 4:**
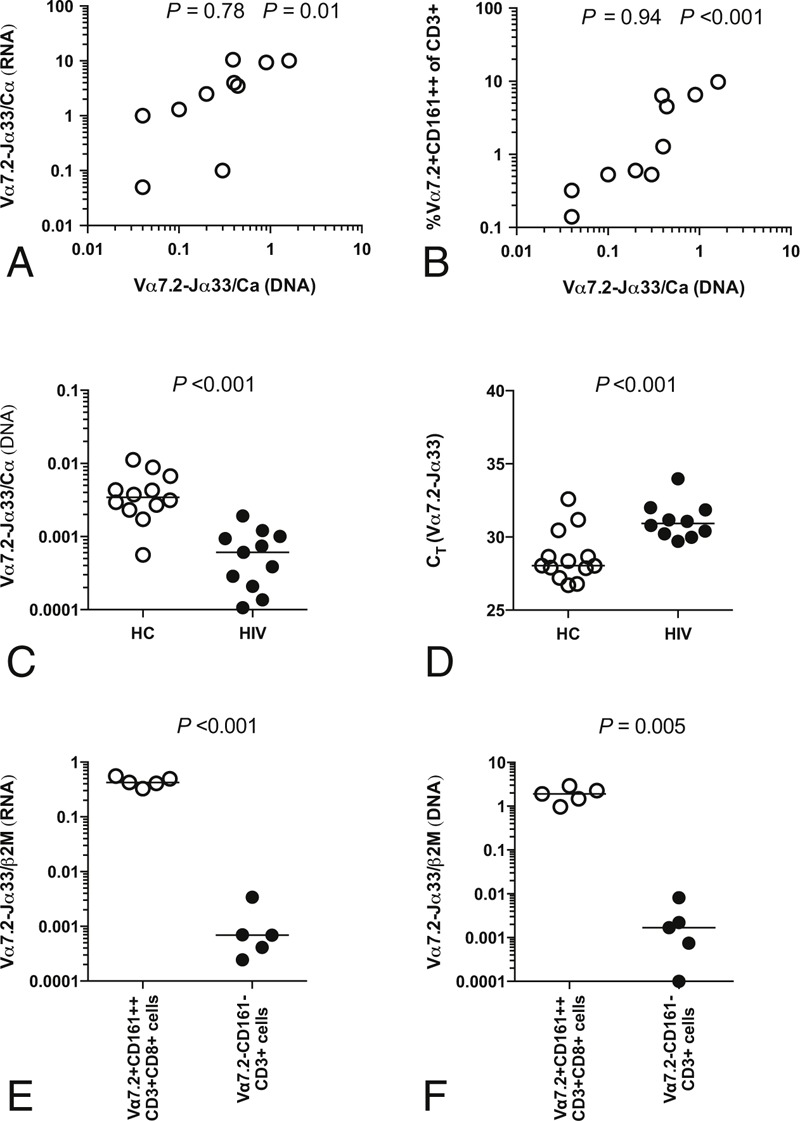
Depletion of Vα7.2-Jα33 gDNA in HIV infection. The relative abundance of Vα7.2-Jα33 gDNA in the PBMCs of HC (n = 10) was correlated with the (A) abundance of Vα7.2-Jα33 mRNA and with the (B) frequency of Vα7.2+CD161++CD3+ cells. Spearman correlations were determined. (C) The relative abundance of Vα7.2-Jα33 gDNA in whole blood of HC (open circles) and HIV-infected patients (filled circles) was compared. (D) C_T_ values for Vα7.2-Jα33 in HC and HIV-infected patients are also shown. The specificity of the PCR was confirmed by assessing the relative abundance of Vα7.2-Jα33 (E) mRNA and (F) gDNA in sorted MAIT cell (Vα7.2+CD161++CD3+CD8+) and non-MAIT cell (Vα7.2−CD161−CD3+) populations. gDNA = genomic DNA, HC = healthy control, MAIT = mucosal-associated invariant T, mRNA = messenger RNA, PBMC = peripheral blood mononuclear cell, PCR = polymerase chain reaction.

As a selective downregulation of MAIT cell TCR expression could account for the apparent decrease in Vα7.2-Jα33 mRNA abundance relative to Cα mRNA in HIV infection, we investigated whether MAIT cell gDNA was also depleted from blood. This is possible because during TCR rearrangement, the variable and junctional regions are joined together to form a single exon.^21^ Using PBMCs from HC, we first confirmed that Vα7.2-Jα33 gDNA abundance correlated with Vα7.2-Jα33 mRNA abundance; as expected, a significant correlation was found (ρ = 0.78, *P* = 0.01) (Figure 4A). Furthermore, Vα7.2-Jα33 gDNA abundance was strongly correlated with the frequency of Vα7.2+CD161++ cells among CD3+ lymphocytes (ρ = 0.94, *P* < 0.001) (Figure 4B). Therefore, Vα7.2-Jα33 gDNA abundance is a valid measure of absolute MAIT cell numbers. We measured the abundance of Vα7.2-Jα33 gDNA in a different cohort of HIV-infected patients and compared it with HC. Consistent with the decreased relative abundance of Vα7.2-Jα33 mRNA (Figure 1), there was a significant decrease in the relative abundance of Vα7.2-Jα33 gDNA (median abundance of the Vα7.2-Jα33 rearrangement relative to Cα 0.00344 vs 0.00061, *P* < 0.001) in HIV+ versus HCs (Figure 4C). As DNA was extracted from equal volumes of blood and equal volumes of extracted DNA were used as the template for PCR, C_T_ could be used as a direct measure of MAIT cell abundance per volume of blood. The C_T_ for Vα7.2-Jα33 was significantly higher (median C_T_ 30.93 vs 28.05, *P* < 0.001) in HIV+ versus HC (Figure 4D), confirming absolute depletion of the MAIT cell population.

To confirm the specificity of the PCR, RNA and DNA were extracted from FACS-sorted MAIT cells (Vα7.2+CD161++CD8+CD3+ lymphocytes) and non-MAIT cells (Vα7.2-CD161−CD3+ lymphocytes). The relative expression of Vα7.2-Jα33 mRNA was 406-fold enriched (Figure 4E) and the Vα7.2-Jα33 gDNA rearrangement was 743-fold enriched (Figure 4F) in the sorted MAIT cell population. These results were consistent with the sort purity (MAIT cells: 94% Vα7.2+CD161++CD3+; non-MAIT cells: <0.01% Vα7.2+CD161++CD3+).

Finally, we assessed whether the relative abundance of Vα7.2-Jα33 mRNA or gDNA correlated with HIV viral load and CD4+ T-cell count. As in our previous study,^13^ no consistent correlation was found (Supplementary Figure 2, http://links.lww.com/MD/A337).

## DISCUSSION

We have confirmed the loss of MAIT cells in blood in HIV by molecular and flow cytometric approaches. In HIV, decreased abundance of both Vα7.2-Jα33 mRNA and gDNA was seen. Abundance of Vα7.2-Jα33 mRNA correlated most strongly with the frequency of Vα7.2+CD161++ cells, irrespective of whether CD4+ T cells were included. Furthermore, no significant increase in frequency of Vα7.2+CD161− cells was seen among CD3+CD4− lymphocytes.

A limitation of our study is that demographic data of HCs were not available. Therefore, age and sex cannot be excluded as confounding variables. It has recently been reported that the frequency of MAIT cells in blood is significantly lower in older (age 61–92 years) than younger (age 21–40 years) individuals.^22^ However, given the low median age of the HIV-infected cohorts in our study (Table 1), age is unlikely to explain the differences observed.

Loss of Vα7.2++CD161++ MAIT cells in HIV infection has previously been reported.^13–16^ One potential mechanism for this is through downregulation of CD161, resulting in a change in phenotype rather than loss of TCR+ cells.^14^ Indeed, in vitro MAIT cells downregulate CD161 upon activation.^14,23^ Given the disrupted intestinal epithelial integrity in HIV infection and the subsequent translocation of bacteria,^13,24–26^ activation of MAIT cells and subsequent downregulation of CD161 would be expected. However, our study suggests that the loss of Vα7.2+CD161++ MAIT cells in HIV infection cannot be solely explained by downregulation of CD161. In HIV-infected patients, the reduced abundance of Vα7.2-Jα33 mRNA and gDNA, the unchanged frequency of Vα7.2+CD161− cells among CD3+CD4− lymphocytes (despite the loss of Vα7.2+CD161++ cells, which would be expected to increase the relative frequency of this population), and the strong correlation of Vα7.2-Jα33 mRNA levels with the frequency of Vα7.2+CD161++ cells but not total Vα7.2+ cells, all argue that MAIT cells are lost from the blood in HIV infection.

Although our data does not exclude the presence of CD161− MAIT cells in blood in HIV infection, 2 recent studies, 1 in healthy donors and 1 in HIV-infected patients, found that MR1-tetramer-positive cells were not present in the CD161− compartment,^10,17^ confirming CD161 as a key marker. Furthermore, despite an increase in frequency of Vα7.2+CD161−CD3+ cells as a percentage of CD3+ cells, no increase was seen in the number of Vα7.2+CD161−CD3+ cells per microliter.^17,18^ This could be further investigated in future studies by quantifying the number of MAIT cell TCR transcripts in flow cytometry-sorted Vα7.2+CD161− cells from the blood of HIV-infected patients.

Activation-induced cell death secondary to microbial translocation and/or sequestration in tissues are alternative explanations that have been suggested for the loss of MAIT cells from the blood in HIV infection.^13,18^ We have previously reported that in the colon, the absolute number of MDR1++CD3+CD8+ T cells, a proxy for MAIT cells, was unchanged in HIV as assessed by immunohistochemistry.^13^ Similarly, no depletion of Vα7.2+CD161++CD3+ cells was seen in rectal biopsies as measured by flow cytometry.^14^ In contrast, it was recently reported that MAIT cells (defined as CD161++CD8+CD3+ cells) were depleted as a proportion of CD8+CD3+ lymphocytes in the colon in HIV, with subsequent recovery after a median of 72 months of antiretroviral therapy.^27^ Interestingly, blood MAIT cell frequencies fail to recover with antiretroviral therapy^13,14^ raising the possibility that recovery occurs first in the colon^27^ and later in the blood. Recovery in blood should therefore be assessed over a longer follow-up period and perhaps also after interventions in very early infection. Furthermore, as the influx of CD8+ T cells into the colon in HIV infection may influence cell proportions,^13,28–30^ thus limiting the effectiveness of flow cytometry, measurement of Vα7.2-Jα33 mRNA or gDNA may provide a sensitive method for measuring MAIT cell dynamics in the tissues during HIV infection and treatment.

A limitation of our study is that our assay does not assess the abundance of Vα7.2-Jα12 and Vα7.2-Jα20 MAIT cells. Although Vα7.2-Jα33 MAIT cells form the overwhelming majority of the blood MAIT cell population in most people, Vα7.2-Jα12 and Vα7.2-Jα20 MAIT cells can comprise up to 50% in some individuals.^10,31^ Vα7.2-Jα12 MAIT cells also appear to be enriched in some tissues, although in most donors Vα7.2-Jα33 MAIT cells still predominate.^31^ Nonetheless, given that Vα7.2-Jα12, Vα7.2-Jα20, and Vα7.2-Jα33 MAIT cell TCRs all bind to ligand-bound MR1,^10^ there is no a priori reason to hypothesize that different subsets are differentially depleted in HIV infection compared with HCs. Therefore, although our analysis only involves a single Jα segment, it is the most abundant in the majority of individuals and the conclusions drawn could reasonably be expected to be applicable to MAIT cells containing alternative Jα segments (Jα12 and Jα20). The fate of alternative MAIT cell populations should be investigated in future studies.

The loss of MAIT cells in HIV may contribute to immunodeficiency. In vivo animal models suggest that MAIT cells have a nonredundant role in controlling systemic and mucosal infection with a range of bacteria, including BCG and *Enterobacteriaceae.*^7,8,32,33^ Given the increased rate of progression to active tuberculosis following infection with *M tuberculosis*^34^ and the high rates of invasive nontyphoidal *Salmonella* infection in HIV infection,^35^ it will be important to determine the role of MAIT cell depletion in HIV-induced immunodeficiency.

In conclusion, through the measurement of Vα7.2-Jα33 mRNA and gDNA levels, we have confirmed that MAIT cells are depleted from the blood in HIV infection and that significant accumulation of a CD161− MAIT cell population is unlikely. Future studies to investigate the fate of MAIT cells in tissue in HIV infection, using these techniques, are warranted.

## Acknowledgments

The authors would like to thank Tabitha Unsworth-White and Emily Adland, University of Oxford, for their assistance with the study, and Andrew Gray, University of Otago, for statistical advice.

Members of the SHCS: Aubert V, Battegay M, Bernasconi E, Böni J, Bucher HC, Burton-Jeangros C, Calmy A, Cavassini M, Dollenmaier G, Egger M, Elzi L, Fehr J, Fellay J, Furrer H (Chairman of the Clinical and Laboratory Committee), Fux CA, Gorgievski M, Günthard H (President of the SHCS), Haerry D (deputy of “Positive Council”), Hasse B, Hirsch HH, Hoffmann M, Hösli I, Kahlert C, Kaiser L, Keiser O, Klimkait T, Kouyos R, Kovari H, Ledergerber B, Martinetti G, Martinez de Tejada B, Metzner K, Müller N, Nadal D, Nicca D, Pantaleo G, Rauch A (Chairman of the Scientific Board), Regenass S, Rickenbach M (Head of Data Centre), Rudin C (Chairman of the Mother & Child Substudy), Schöni-Affolter F, Schmid P, Schüpbach J, Speck R, Tarr P, Telenti A, Trkola A, Vernazza P, Weber R, Yerly S (http://www.shcs.ch/184-for-shcs-publications).
